# An Improved Long-Period Precise Time-Relative Positioning Method Based on RTS Data

**DOI:** 10.3390/s21010053

**Published:** 2020-12-24

**Authors:** Yangwei Lu, Shengyue Ji, Rui Tu, Duojie Weng, Xiaochun Lu, Wu Chen

**Affiliations:** 1 National Time Service Center, Chinese Academy of Sciences, Shu Yuan Road, Xi’an 710600, China; luyangwei@ntsc.ac.cn (Y.L.); turui@ntsc.ac.cn (R.T.); luxc@ntsc.ac.cn (X.L.); 2 Department of Surveying and Mapping, China University of Petroleum (East China), Qingdao 266580, China; 19990045@upc.edu.cn; 3 Shenzhen Research Institute, The Hong Kong Polytechnic University, Shenzhen 518057, China; wu.chen@polyu.edu.hk; 4 Department of Land Surveying and Geo-Informatics, The Hong Kong Polytechnic University, Hong Kong 999077, China

**Keywords:** GNSS, satellite receivers, positioning error, positioning algorithm

## Abstract

The high precision positioning can be easily achieved by using real-time kinematic (RTK) and precise point positioning (PPP) or their augmented techniques, such as network RTK (NRTK) and PPP-RTK, even if they also have their own shortfalls. A reference station and datalink are required for RTK or NRTK. Though the PPP technique can provide high accuracy position data, it needs an initialisation time of 10–30 min. The time-relative positioning method estimates the difference between positions at two epochs by means of a single receiver, which can overcome these issues within short period to some degree. The positioning error significantly increases for long-period precise positioning as consequence of the variation of various errors in GNSS (Global Navigation Satellite System) measurements over time. Furthermore, the accuracy of traditional time-relative positioning is very sensitive to the initial positioning error. In order to overcome these issues, an improved time-relative positioning algorithm is proposed in this paper. The improved time-relative positioning method employs PPP model to estimate the parameters of current epoch including position vector, float ionosphere-free (IF) ambiguities, so that these estimated float IF ambiguities are used as a constraint of the base epoch. Thus, the position of the base epoch can be estimated by means of a robust Kalman filter, so that the position of the current epoch with reference to the base epoch can be obtained by differencing the position vectors between the base epoch and the current one. The numerical results obtained during static and dynamic tests show that the proposed positioning algorithm can achieve a positioning accuracy of a few centimetres in one hour. As expected, the positioning accuracy is highly improved by combining GPS, BeiDou and Galileo as a consequence of a higher amount of used satellites and a more uniform geometrical distribution of the satellites themselves. Furthermore, the positioning accuracy achieved by using the positioning algorithm here described is not affected by the initial positioning error, because there is no approximation similar to that of the traditional time-relative positioning. The improved time-relative positioning method can be used to provide long-period high precision positioning by using a single dual-frequency (L1/L2) satellite receiver.

## 1. Introduction

High accuracy real-time positioning is the most important application of Global Navigation Satellite System (GNSS). In recent years, the redundancy and diversity of satellites comprising the space segment of GNSS have significantly increased, so that the reliability and availability of positioning systems are highly enhanced. At the same time, this also contributes to the complication of GNSS data processing techniques, due to the various sources of positioning error [1,2,3,4,5].

Nowadays, high-precision positioning can be easily achieved by using real-time kinematic (RTK) and precise point positioning (PPP) or their augmented techniques, such as network RTK (NRTK) and PPP-RTK [6,7]. Traditional RTK or NRTK requires a reference station and datalink in order to minimise the various sources of positioning error, including satellite orbit error (ephemeris), satellite clock error, ionospheric and tropospheric delays, as well as receiver clock error. As the double-differenced (DD) ionospheric and tropospheric delay increases over the distance between base station and mobile receiver, this would decrease the positioning accuracy and increase the (re-)initialisation time of RTK [8]. Though the NRTK overcomes these issues to some degree, a much higher amount of reference stations are required, while a stable and efficient central data processing software must be developed.

The PPP technique can provide high absolute positioning accuracy as consequence of precise orbits and clocks by means of a single receiver. For real-time applications, the precise satellite orbits are also provided by many governmental bodies and manufacturers of satellite receivers. One of the open issues is that the PPP technique has a long initialisation and re-initialisation time. Generally, it is about 10–30 min, in order to achieve centimetre-level positioning accuracy [9], which limits the high accuracy real-time positioning applications. Although the augmented PPP technique, based on different GNSS and using a dual-frequency satellite receiver, together with local reference stations, significantly shortens the above time to first point [10,11], a local reference station and datalink are necessary. Furthermore, much attention should be paid to ambiguity resolution and validation, which affects the positioning reliability for both RTK and PPP.

A time-relative positioning algorithm able to compute the time-differenced carrier phase (TDCP) is proposed in this paper, in order to eliminate the processing ambiguities if there is no cycle slip [12,13,14,15].

According to the difference of data processing techniques, the time-relative positioning method can be divided into two categories: the overall time-relative positioning [15] and accumulated time-relative positioning [16], both of which meet the needs of the two aforementioned precise positioning techniques. Furthermore, both of them can be used to determine user position by using a single receiver without (re-)initialisation time. For these advantages, time-relative positioning has been widely used in many fields, such as engineering surveying, mapping [17], navigation [18,19,20,21,22], altitude determination [15,23] and geological monitoring [24,25].

However, both the above categories of time-relative positioning have also drawbacks. In fact, as the various sources of positioning error remained in TDCP solutions, the positioning accuracy of accumulated time-relative method significantly decreases over the integration, due to the error sources themselves. It is difficult to achieve centimetre-level accuracy by using long-period positioning [17]. The positioning accuracy would be comparable for traditional RTK and overall time-relative positioning if the related errors caused by the different sources (satellite orbit and clock error, ionospheric and tropospheric delays) are constant over time [14].The errors caused by different sources are variable over time, so that the positioning accuracy decreases when the time between the base epoch and the current positioning one becomes longer [13]. The positioning error increases almost linearly over time within short period (linearization error), so that the position data can be corrected through the linear interpolation [26]. Therefore, this assumption of linear drift of the positioning error limits the application of the above methods to a few minutes. If the positioning period is longer than a few minutes, this drift cannot be fully removed through linear interpolation.

Furthermore, both of these methods are sensitive to the initial position [17,19]. In fact, as the initial position induces the above linearisation error of position data for the two epochs (base and current ones), the positioning accuracy may decrease. Different from accumulated time-relative positioning method, the integration process is not needed for overall time-relative positioning method. If the sources of positioning error are properly minimized for long-period precise positioning, the positioning accuracy achieved by using the overall time-relative positioning method is expected to be much higher than that obtained by means of the accumulated time-relative method.

This work is aimed at improving the positioning accuracy of the overall time-relative method for long-period precise positioning. After analysing the main error sources of the time-relative positioning, an improved multi-GNSS time-relative positioning algorithm, based on ionosphere-free (IF) combination measurements and real-time service (RTS) data stream, is proposed. The proposed time-relative positioning method, which is not affected by the initial position error, is able to achieve a positioning accuracy of a few centimeters in one hour. Thus, the positioning accuracy is assessed during long-time static and dynamic tests.

## 2. Time-Relative Positioning Algorithm Based on TDCP Measurements

### 2.1. Mathematical Models of Time-Relative Positioning Method

In order to achieve time-relative positioning, GNSS measurements were firstly recorded at base epoch $T_{b}$, then the receiver was moved to another station i at epoch $T_{i}$ to collect GNSS data for a short session. The measurement equations of the two sessions can be read as follows:

(1) $$\{\begin{array}{c} L_{b}=A_{b}X_{b}+BN+cdt_{b}^{r}+d_{b}^{orb}-cdt_{b}^{s}+T_{b}-I_{b}\\ L_{i}=A_{i}X_{i}+BN+cdt_{i}^{r}+d_{i}^{orb}-cdt_{i}^{s}+T_{i}-I_{i} \end{array}$$

where: $L_{b}$ and $L_{i}$ are the carrier phase measurements for epoch $T_{b}$ and $T_{i}$; $X_{b}$ and $X_{i}$ are the position vectors of epoch $T_{b}$ and $T_{i}$, respectively; N is ambiguity vector; $A_{b}$, $A_{i}$ and B are corresponding matrix coefficients; $d^{orb}$ is the satellite orbit error; $dt^{r}$ is the receiver clock error; $dt^{s}$ is the satellite clock error; T is the tropospheric delay, I is the ionospheric delay.

Time-differencing can be formed between $T_{b}$ and $T_{i}$ epochs, so that, it is possible to obtain:

(2) $$\begin{array}{l} \;L_{i}-L_{b}-\left(\left(d_{i}^{orb}-d_{b}^{orb}\right)-c\left(dt_{i}^{s}-dt_{b}^{s}\right)\right)\\ =\left(A_{i}X_{i}-A_{b}X_{b}\right)+c\left(dt_{i}^{r}-dt_{b}^{r}\right)+\left(T_{i}-T_{b}\right)-\left(I_{i}-I_{b}\right) \end{array}$$

Because the time-differenced measurement is not the direct function of relative position $X_{b,i}$ (position relative to base epoch) [16], the Equation (2) is usually solved for $X_{i}$. Thus, the Equation (2) can be rewritten as:

(3) $$\begin{array}{c} L_{i}-L_{b}-d_{b,i}^{orb}+cdt_{b,i}^{s}\\ =\left(A_{i}X_{i}-A_{i}X_{b}+A_{i}X_{b}-A_{b}X_{b}\right)+cdt_{b,i}^{r}+T_{b,i}-I_{b,i}\\ =\left(A_{i}dX_{b,i}+dA_{bi}X_{b}\right)+cdt_{b,i}^{r}+T_{b,i}-I_{b,i} \end{array}$$

The difference of ionospheric and tropospheric delays between the two epochs can be neglected for short-time positioning after correcting with the algorithm, while the ionospheric delays are eliminated with IF combination. The satellite orbit and clock are constrained with the forecast ephemeris or real-time precise orbit and clock. Thus, the time-differential relative positioning model of the two epochs is formed as: $dL_{b,i}=A_{i}dX_{b,i}+dA_{b,i}X_{b}+cdt_{b,i}^{r}$.

If the interval between $T_{b}$ and $T_{i}$ is enough, the difference between $A_{b}$ and $A_{i}$ can be neglected, thus:

(4) $$A_{i}dX_{b,i}+cdt_{b,i}^{r}=dL_{b,i}$$

If the position of the base epoch $T_{b}$ is known, then the coordinates of station and clock parameter can be estimated based on least squares with more than four satellites.

### 2.2. Error Analysis

The time-relative positioning error is mainly caused by the temporal, between $T_{b}$ and $T_{i}$ epochs, of the errors caused by the different sources, including those in satellite orbits and satellite clocks, ionospheric and tropospheric delays, as well as initial positioning error, which cannot be removed through time-differencing for long-period precise positioning.

The error affecting forecast ephemeris is variable over time, so that it cannot be removed through time-differencing. Furthermore, the orbital error rapidly increases. For example, the 3D orbital error can increase up to 70 cm in 30 min for GPS. The error affecting GPS satellite clock increases slightly slower than that of the forecast ephemeris and the magnitude is about 7–30 cm in 30 min [27]. Furthermore, the error affecting the broadcast ephemeris cannot be fitted with the linear model for long period. The remaining error decreases the positioning accuracy over the interval of two epochs for time-relative positioning.

Fortunately, the International GNSS Service (IGS) RTS has been providing access to continuous real-time GNSS forecast ephemeris and clock corrections since the 1 April 2013 [28,29]. The accuracy of RTS products is about 8 cm for GPS ephemeris and 0.3 ns for GPS satellite clocks [28,30], which is enough high for real-time precise positioning. As the real-time high-rate precise products are provided, the orbital and clock errors would not increase over time.

The ionospheric delay is the main error in time-relative positioning for single-frequency satellite receiver (L1, i.e., 1575.42 MHz): the magnitude would increase up to 128 cm in 30 min [27]. As the change rate of ionospheric delay is variable with the site and the epoch, it is difficult to precisely model it. Furthermore, the change rate of ionospheric delay is easily influenced by space weather, its value would be very high and most of the ionosphere models also perform badly during high solar activity period [31]. While for dual-frequency satellite receivers (L1/L2, i.e., 1227.60 MHz), the IF combination can be formed to minimize the ionospheric delay.

The tropospheric delay consists of two parts: the hydrostatic delay and the wet delay [32]. The tropospheric delay changes much slower than ionospheric delay, so that most of them can be removed through time-differencing within a short period. However, both of the hydrostatic and wet delays are heavily dependent on the elevation mask, and the remaining error after time-differencing would increase over the time interval between base epoch and current one. The hydrostatic delay can be accurately modelled based on the information of atmospheric pressure [33], while the wet delay is difficult to model and predict. The wet delay is usually estimated as a random walk process with GNSS measurements [34,35].

Another important error source is the initial positioning error. As described in the previous paragraph, the difference between *A_b_* and $A_{i}$ is neglected. Furthermore, the errors affecting the base epoch coordinates will also increase the positioning error when geometrical satellite distribution changes, especially for long-period precise positioning.

In conclusion, the main factors that limit the time-relative positioning accuracy for long-period positioning include errors in forecast ephemeris, ionospheric delay, tropospheric delay and the error of initial position. According to the above analysis, an improved time-relative positioning algorithm is proposed in this paper, as well as the mathematic model and performance are here presented.

## 3. Improved Time-Relative Positioning Algorithm for Long-Period Precise Positioning

In order to minimise the errors analysed in the previous paragraph, an improved overall time-relative positioning algorithm is proposed for long-period precise positioning.

As the errors affecting forecast ephemeris are accumulated with the increase of time between two epochs, the real-time precise satellite orbits and clocks are used in this paper for their high accuracy and time-resolution, which is expected to minimise most of the errors in forecast ephemeris. In order to reduce the effect of ionospheric delay, the IF combination measurements are employed to estimate the related parameters. Thus, the remaining errors are the tropospheric delay and the error induced by the initial position.

The proposed time-relative positioning algorithm consists of two steps. Firstly, the PPP solution is obtained with IF combination based on RTS products, then the estimated IF float ambiguities are used as constraints to estimate the user position, tropospheric delay and receiver clock error. Finally, the time-relative positioning results are calculated by differencing the coordinates of current epoch and the base one, and the mathematical models are described as follows.

### 3.1. PPP Model Used with RTS Products

The GNSS code and carrier phase IF combinations read [36]:

(5) $$\{\begin{array}{c} P_{IF,r}^{m,s}=\frac{1}{f_{m,1}^{2}-f_{m,2}^{2}}\left(f_{m,1}^{2}P_{1,r}^{m,s}-f_{m,2}^{2}P_{2,r}^{m,s}\right)\\ L_{IF,r}^{m,s}=\frac{1}{f_{m,1}^{2}-f_{m,2}^{2}}\left(f_{m,1}^{2}L_{1,r}^{m,s}-f_{m,2}^{2}L_{2,r}^{m,s}\right) \end{array}$$

where: m, r and s indicate the satellite system, receiver and satellite; $P_{IF,r}^{m,s}$ and $L_{IF,r}^{m,s}$ are the IF combination measurements for pseudorange and carrier phase.

The IF PPP function model for GPS, Galileo and BeiDou can be presented as [37]:

(6) $$\{\begin{array}{l} P_{IF,r}^{G,s}=\rho_{r}^{G,s}+d_{orb}^{G}-c*dt^{G,s}+c*dt_{r}^{G}+m_{r}^{G,s}*T+\varepsilon (P_{IF,r}^{G,s})\\ L_{IF,r}^{G,s}=\rho_{r}^{G,s}+d_{orb}^{G}-c*dt^{G,s}+c*dt_{r}^{G}+m_{r}^{G,s}*T+B_{IF,r}^{G,s}+\varepsilon (L_{IF,r}^{G,s}) \end{array}$$

(7) $$\{\begin{array}{l} P_{IF,r}^{E,s}=\rho_{r}^{E,s}+d_{orb}^{E}-c*dt^{E,s}+c*dt_{r}^{G}+c*dt_{sys}^{E}+m_{r}^{E,s}*T+\varepsilon (P_{IF,r}^{E,s})\\ L_{IF,r}^{E,s}=\rho_{r}^{E,s}+d_{orb}^{E}-c*dt^{E,s}+c*dt_{r}^{G}+c*dt_{sys}^{E}+m_{r}^{E,s}*T+B_{IF,r}^{E,s}+\varepsilon (L_{IF,r}^{E,s}) \end{array}$$

(8) $$\{\begin{array}{l} P_{IF,r}^{C,s}=\rho_{r}^{C,s}+d_{orb}^{C}-c*dt^{C,s}+c*dt_{r}^{G}+c*dt_{sys}^{C}+m_{r}^{C,s}*T+\varepsilon (P_{IF,r}^{C,s})\\ L_{IF,r}^{C,s}=\rho_{r}^{C,s}+d_{orb}^{C}-c*dt^{C,s}+c*dt_{r}^{G}+c*dt_{sys}^{C}+m_{r}^{C,s}*T+B_{IF,r}^{C,s}+\varepsilon (L_{IF,r}^{C,s}) \end{array}$$

where: G, E and C indicate the GNSS index (G: GPS, E: Galileo and C: BeiDou); $dt_{r}^{G}$ represents the receiver clock error of GPS; $dt_{sys}^{E}$ and $dt_{sys}^{C}$ refer to the GNSS time difference parameters for Galileo and BeiDou with reference to GPS, respectively. P and L are the pseudorange and carrier phase, respectively, and ρ is the geometrical distance between receiver and satellite (range). T denotes the zenith tropospheric delay, m indicates the troposphere mapping function; B is the corresponding IF float ambiguity; ε stands for the measurement noise; c represents the speed of light in vacuum, $d_{orb}^{G}$ refers to the satellite orbital error, $dt^{G,s}$ is the satellite clock error.

The satellite orbits and clocks are constrained with RTS products, as well as a robust Kalman filter is used to estimate the related parameters, including coordinates, receiver clock error, the positioning error of Galileo/BeiDou with reference to GPS, zenith tropospheric wet delay and ambiguities.

### 3.2. Time-Relative Positioning with the Constraints of IF Float Ambiguities

Supposing that the first epoch $t_{0}$ is the base epoch, then the 3D positions $(X_{x}\left(t_{i}\right)$, $X_{y}\left(t_{i}\right)$, $X_{z}\left(t_{i}\right))$ and IF float ambiguities $(B_{IF,r}^{G,s1}\left(t_{i}\right)$, …, $B_{IF,r}^{G,sn}\left(t_{i}\right)$, $B_{IF,r}^{E,s1}\left(t_{i}\right)$, …, $B_{IF,r}^{E,sn}\left(t_{i}\right)$, $B_{IF,r}^{C,s1}\left(t_{i}\right)$, …, $B_{IF,r}^{C,sn}\left(t_{i})\right)$ of epoch $t_{i}$ can be estimated by using the PPP method based on RTS products. The IF float ambiguities of all epochs should be similar for the same satellite if all the cycle slips are detected and repaired. Thus, the estimated IF ambiguities can be used as constraints for the base epoch, in order to estimate other parameters, including 3D positions, receiver clock error and tropospheric delay. In this paper, the cycle slips are detected and repaired by using the Melbourne–Wübbena and geometry-free combination method [38,39].

Applying IF ambiguities constraint functionally implies fixing the ambiguities parameters in the carrier phase function model. Equation (9) illustrates the function models with the IF float ambiguities to be constrained:

(9) $$\{\begin{array}{l} P_{r}^{s}=\rho_{r}^{s}+d_{orb}-c*dt^{s}+c*dt_{r}+m_{r}^{s}*T+\varepsilon (P_{r}^{s})\\ L_{r}^{s}=\rho_{r}^{s}+d_{orb}-c*dt^{s}+c*dt_{r}+m_{r}^{s}*T+B_{r,constrained}^{s}+\varepsilon (L_{r}^{s}) \end{array}$$

where: $B_{r,constrained}^{s}$ is the constrained IF float ambiguities. For the base epoch, the IF float ambiguities are constrained as:

(10) $$\{\begin{array}{c} B_{IF,r}^{G,s1}\left(t_{0}\right)=B_{IF,r}^{G,s1}\left(t_{i}\right)\\ \vdots \\ B_{IF,r}^{G,sn}\left(t_{0}\right)=B_{IF,r}^{G,sn}\left(t_{i}\right)\\ B_{IF,r}^{E,s1}\left(t_{0}\right)=B_{IF,r}^{E,s1}\left(t_{i}\right)\\ \vdots \\ B_{IF,r}^{E,s1}\left(t_{0}\right)=B_{IF,r}^{E,s1}\left(t_{i}\right)\\ B_{IF,r}^{C,s1}\left(t_{0}\right)=B_{IF,r}^{C,s1}\left(t_{i}\right)\\ \vdots \\ B_{IF,r}^{C,s1}\left(t_{0}\right)=B_{IF,r}^{C,s1}\left(t_{i}\right) \end{array}$$

As previously shown, the IF float ambiguities are being continuously estimated through the traditional PPP model, then the estimated IF ambiguities of the current epoch are used as a constraint to estimate the position for the base epoch (first epoch). The relative position is calculated by differencing the positions of current and base epochs. Based on this method, the positioning error can be removed by time-differencing within a short period. For long-period positioning, the IF ambiguities, which can be used as a constraint for the base epoch, tend to converge towards the true value.

By constraining the IF ambiguities parameters in the function models, the corresponding derivatives presented in the design matrix must be deleted. The constrained parameters are not necessary to be estimated through this method, while there is potential error in the solution, due to the error in the estimated IF float ambiguities. Another option is implementing stochastic constraints, which are much more robust and simpler. Based on the IF ambiguities estimated and the uncertainties of those ambiguities, the covariance matrix can be adjusted to constrain the IF ambiguities.

The stochastic constraints (the covariance between these parameters is ignored) for the base epoch can be read as:

(11) $$Q_{x^{0}}=[\begin{array}{ccccccc} \sigma_{x}^{2} & & & & & & \\ & \sigma_{y}^{2} & & & & & \\ & & \sigma_{z}^{2} & & & & \\ & & & \ddots & & & \\ & & & & \sigma_{IF,sys}^{2,s1} & & \\ & & & & & \ddots & \\ & & & & & & \sigma_{IF,sys}^{2,sn} \end{array}]$$

where: $Q_{x^{0}}$ is a priori variance-covariance matrix, $\sigma_{x}^{2}$, $\sigma_{y}^{2}$, $\sigma_{z}^{2}$ are a priori variance-covariance of coordinates; $\sigma_{IF,sys}^{2,s1}$ is the constrain uncertainty of the estimated IF float ambiguities; sys indicates different GNSS systems.

The choice of uncertainties would affect the performance of the base epoch. In order to avoid the case of overconstraining, the variance of the estimated IF float ambiguities is determined as follows [40]:

(12) $$\{\begin{array}{c} \sigma_{IF,G}^{s1}=0.0008m\\ \sigma_{IF,E}^{s1}=0.0008m\\ \sigma_{IF,C}^{s1}=0.0010m \end{array}$$

Then, the coordinates and the parameters of the base epoch can be estimated by means of a robust Kalman filter. Thus, the positioning related to the base epoch $t_{0}$ can be calculated as follows:

(13) $$dX_{b,i}=X\left(t_{i}\right)-X\left(t_{0}\right)$$

The new time-relative positioning method estimates the position both for base and current epochs, differently from traditional TDCP positioning, using approximation for determining positions. Meanwhile, all the errors caused by the different sources in GNSS measurements are precisely computed, so that a high positioning accuracy is expected to be provided by long-period positioning, based on the proposed new algorithm.

**Figure 1:**
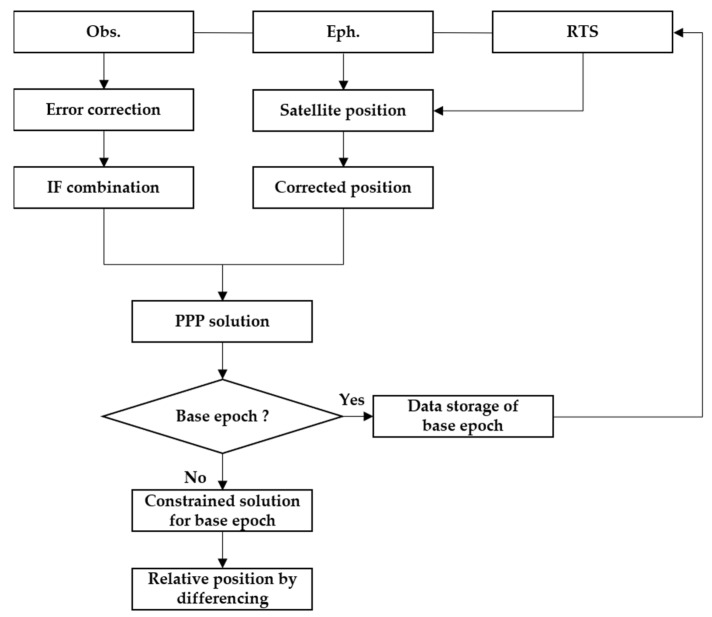
Flowchart of the proposed new algorithm.

The flowchart of the proposed algorithm is shown in Figure 1. The steps of the algorithm can be summarised as follows:

(1) Calculation of satellite position by using the forecast ephemeris and its correction by means of RTS data;
(2) Estimation of IF float ambiguities and user position by means of the robust Kalman filter based on the PPP model;
(3) Estimation of user position by constraining the IF float ambiguities through the robust Kalman filter for the base epoch; the stochastic constraints are adopted in this work;
(4) Calculation of the relative position of the current epoch, related to the base epoch, by differencing the estimated position of both epochs.

## 4. Experiments and Results

The real-time positioning examples were used to assess the positioning accuracy of the described algorithm. In order to investigate the positioning accuracy of the new positioning method for long period precise positioning, two data sets sensed by means of dual-frequency (L1/L2) satellite receivers, used with a position update rate of 1 Hz during static and dynamic tests, were analysed.

**Figure 2:**
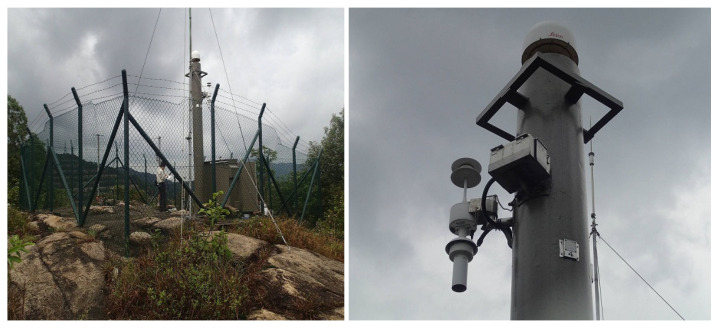
Photographs of the HKLT (Hong Kong Lam Tei) station obtained from the website of Geodetic Survey of Hong Kong.

During static tests, the GNSS data transmitted every hour for 24 h by the station HKLT (Hong Kong Lam Tei, ftp://ftp.geodetic.gov.hk/rinex3/2019/, the location was shown in Figure 2) to a Trimble R9 receiver in Hong Kong on 3 March 2019 were processed through kinematic mode. The static PPP solutions determined by using IGS precise satellite orbital position and clock times were used as reference data. The dynamic data were sensed by means of a Trimble R10 receiver in Qingdao on the 23 September 2019, during one-hour GPS/Galileo/BeiDou measurements. The satellite products containing GPS/ Galileo/BeiDou orbits and clocks corrections obtained from CNT, a product of the Centre National d’Etudes Spatiales (CNES), were used.

**Figure 3:**
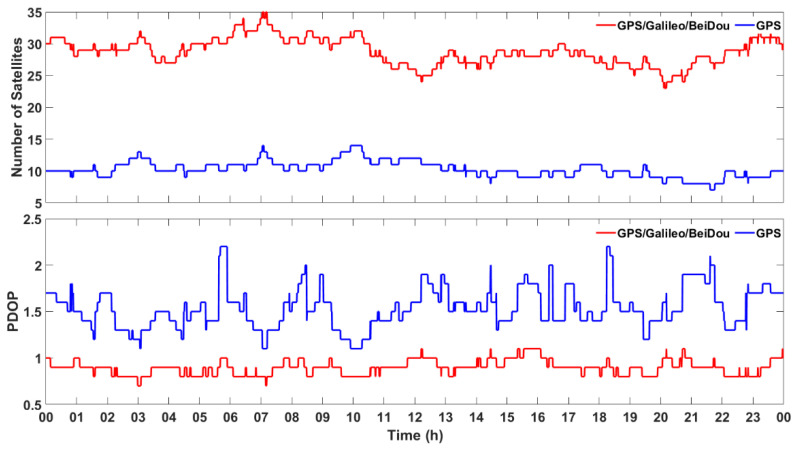
Number of visible satellites (**Top**) and positional dilution of precision (PDOP; **Bottom**) obtained at HKLT station by using only GPS satellites and GPS/Galileo/BeiDou ones, respectively.

Figure 3 shows the number of visible satellites (having an elevation mask higher than 8°) and PDOP (positional dilution of precision) at HKLT station on the 3 March 2019, both for GPS and the combination of GPS, Galileo and BeiDou. There were about 10 GPS visible satellites in each epoch, even if the total number of visible GPS, Galileo and BeiDou satellites could even reach 35, which resulted in much smaller PDOP. Such a redundancy significantly increased the reliability and robustness of precise positioning.

**Figure 4:**
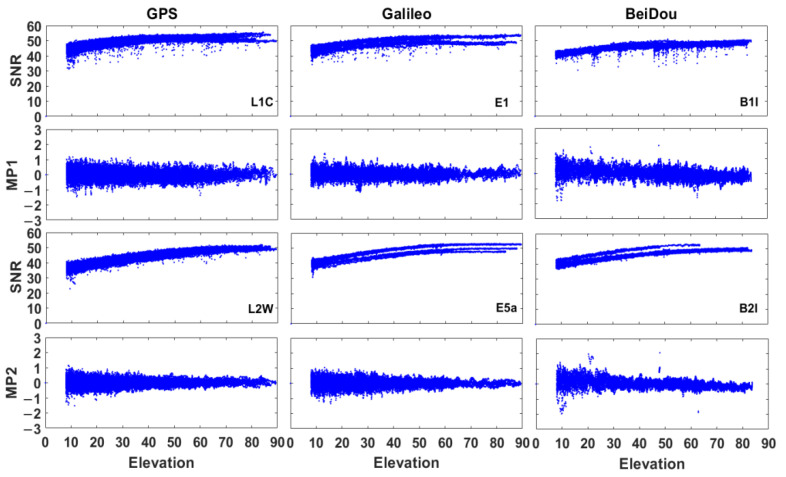
Signal-to-noise ratio (SNR, expressed as dB-Hz) and pseudorange multipath (MP1 and MP2, expressed as metres, the elevation was expressed in degrees) at HKLT station by using GPS, Galileo and BeiDou satellites.

The signal-to-noise ratio (SNR) and pseudorange multipath (MP1/MP2) by using GPS, Galileo and BeiDou satellites were shown in Figure 4. The GNSS measurements were affected by interferences (such as multipath errors) as shown in Figure 4, even though the HKLT station is a continuous reference station.

**Figure 5:**
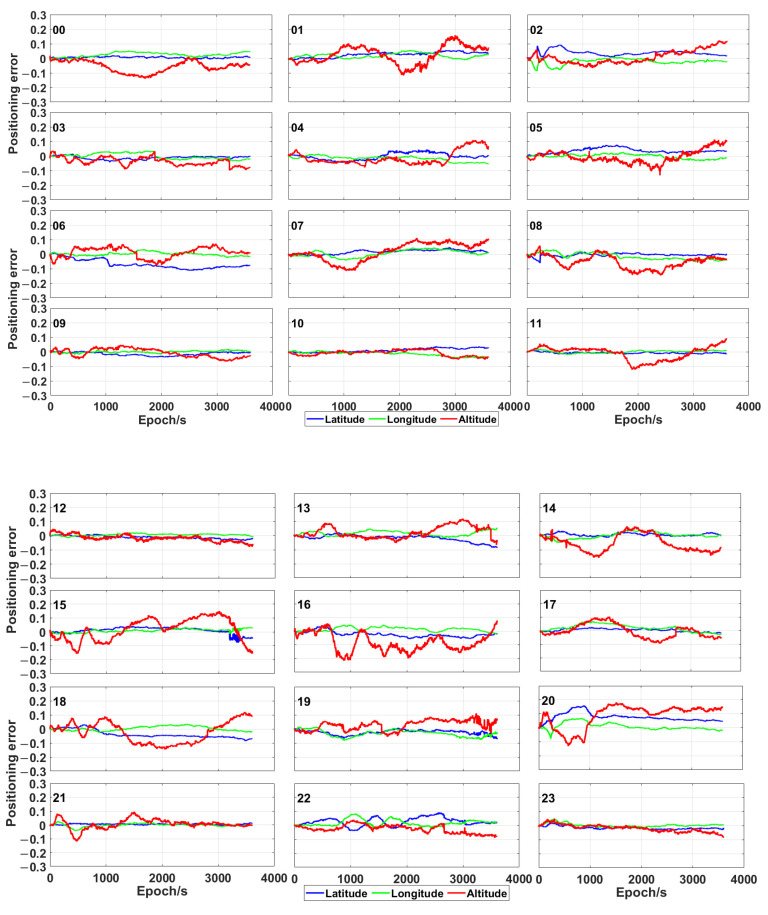
Positioning error (expressed in metres) obtained by means of the proposed time-relative positioning method by using only GPS at different hours.

**Figure 6:**
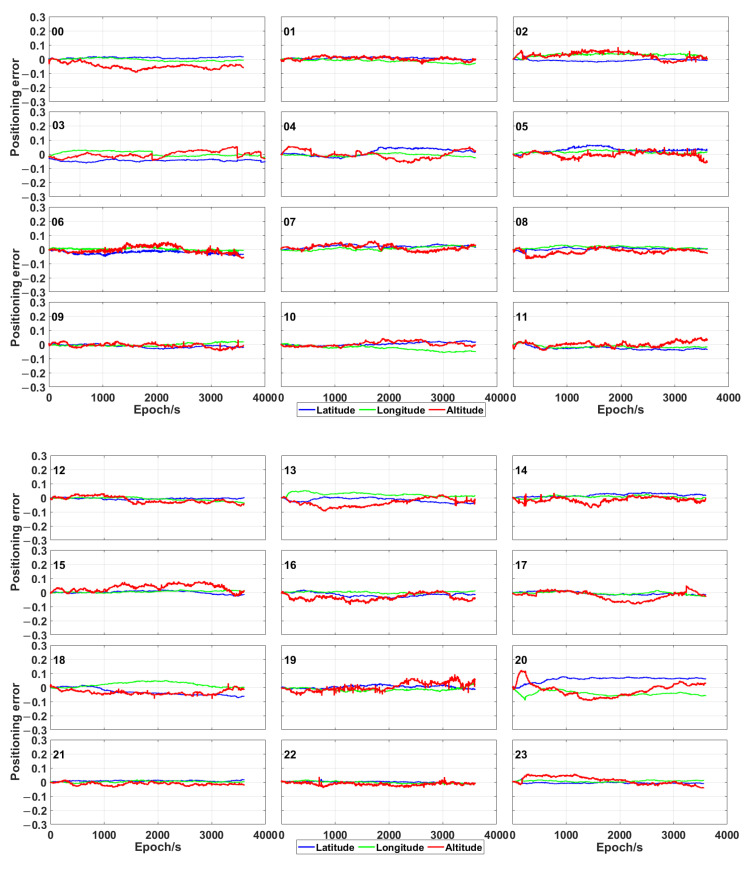
Positioning error (expressed in metres) obtained by means of the proposed time-relative positioning method by using GPS, Galileo and BeiDou at different hours.

The plots in Figure 5 and Figure 6 show the positioning error of the proposed time-relative positioning method (the initial coordinates are estimated by using single point positioning method) for HKLT station by using only GPS satellites and GPS/Galileo/BeiDou ones, respectively. As shown in Figure 5 and Figure 6, the positioning error was uniformly distributed during each hour. There was no significant increase or decrease trend of the positioning errors related to longitude, latitude and altitude during each hour. Moreover, the 3D positioning error fluctuated up to 0.2 m during most sessions using GPS only. As expected, the positioning accuracy was significantly improved by using the combination of GPS, Galileo and BeiDou, as consequence of a higher amount of measurements and a more uniform geometrical distribution of the satellites (expressed by a lower PDOP in Figure 3). The positioning error was lower than 0.1 m for the combination of GPS, Galileo and BeiDou.

**Figure 7:**
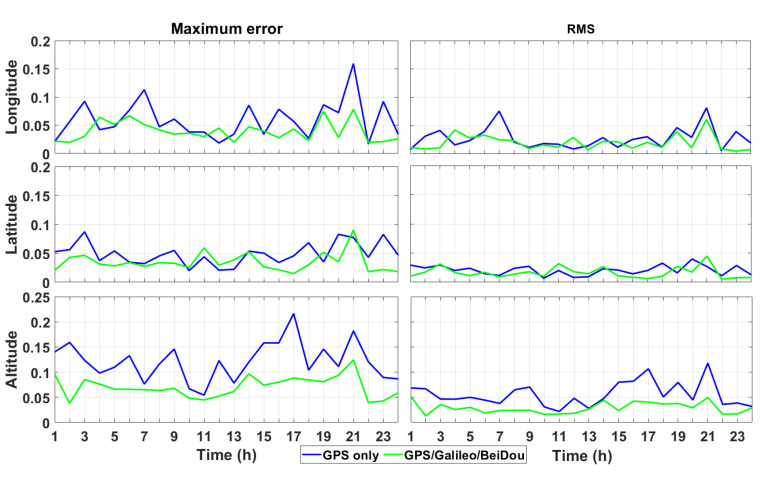
Statistical results (both of the maximum error and RMS were expressed in metres) of the proposed time-relative positioning method obtained by using only GPS and GPS/Galileo/BeiDou.

Table 1 and Table 2 show the statistical results of the positioning accuracy for the 24 h session’s hourly measurements: mean; standard deviation (STD); root mean square (RMS). Figure 7 shows the maximum positioning error and error, expressed as RMS, for each session. This figure shows that the proposed positioning model achieved a positioning error (expressed as RMS) of a few centimetres and a maximum positioning error of 0.22 m.

Furthermore, the mean values of the positioning error fluctuated around zero and most of them fluctuate up to 2 cm, by using both only GPS and the combination of GPS/Galileo/BeiDou. The standard deviation of most sessions was very close to the positioning error, expressed as RMS. Therefore, the positioning accuracy was uniformly distributed by using the proposed positioning method.

For the measurements using only GPS, the horizontal positioning error was up to 0.2 m and, for most of them, it fluctuated up to 0.05 m, while the vertical positioning error was up to 0.25 m and, for most of these measurements, was lower than 0.1 m. As a consequence, the horizontal positioning error, expressed as RMS, fluctuated around 0.03 m, while the vertical positioning error fluctuated around 0.05 m. By comparison with the results obtained by using only GPS, the positioning accuracy was highly improved by means of the combination of GPS, Galileo and BeiDou. In fact, both the maximum positioning error and that expressed as RMS were much lower than those obtained by using only GPS: the horizontal positioning error was about 0.02 m, while the vertical one was 0.04 m.

**Figure 8:**
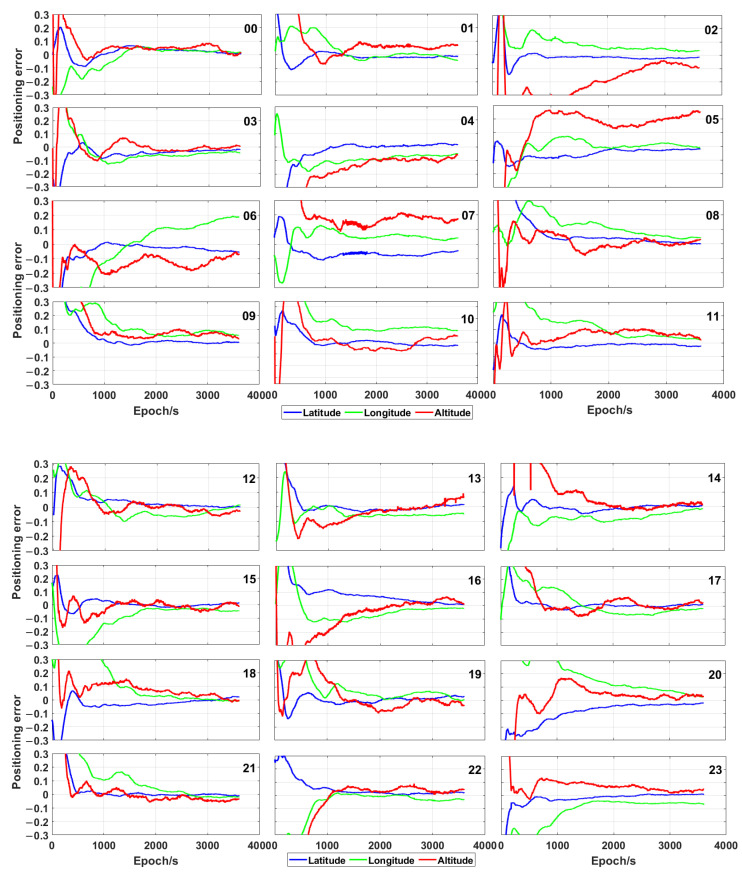
Precise point positioning (PPP) error (expressed in metres) obtained by using GPS/Galileo/BeiDou satellites and CNT (a product of the Centre National d’Etudes Spatiales) at different times.

In order to validate the developed algorithm, the traditional real-time PPP position data calculated by means of GAMP (GNSS Analysis software for Multi-constellation and multi-frequency Precise positioning) [41] by using GPS/Galileo/BeiDou satellites in 24 h are plotted in Figure 8. The initialisation time is inevitable for every session and is more than 10 min (the positioning errors were lower than 10 cm) for most sessions, even by using the combination of GPS, Galileo and BeiDou. Furthermore, the positioning error fluctuated a lot and was much higher than that obtained by using the proposed time-relative method. Thus, the positioning accuracy of the new algorithm is higher than that of the traditional real-time PPP technique in the time of 1 h.

As the approximate initial position accuracy also affects the traditional time-relative positioning accuracy [19], a data set having different initial position errors (i.e., by adding errors of 5, 10 and 20 m, respectively, to each geodetic coordinate) was tested in one hour (00:00–01:00), in order to confirm whether the proposed algorithm in this work can overcome this issue.

**Figure 9:**
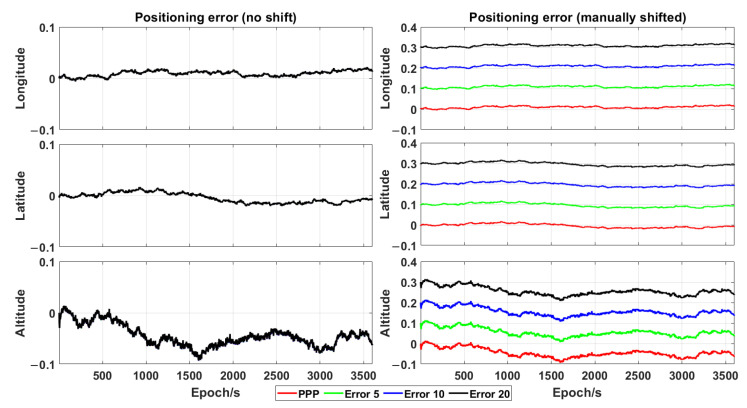
Positioning error (expressed in metres) vs. initial position errors at HKLT station (the red curve comprises the position data obtained by relying on the initial position sensed by means of a static PPP, while the green, blue and black curves are the position data manually shifted by 0.1, 0.2 and 0.3 m, respectively, from the initial position having additional errors of 5, 10 and 20 m, respectively).

Figure 9 shows the positioning error as a function of the initial position error. The reference position data (red curve in Figure 9) were obtained by relying on the initial position sensed by means of a static PPP. Then, errors of 5, 10 and 20 m were added to this initial position. By comparison with the reference position data, there was no significant decrease of positioning accuracy (left panel in Figure 9), based on the initial position having a positioning error of 10 m. In fact, the position was estimated for both the base and current epochs and there was no approximation, differently from the traditional time-relative positioning.

Therefore, the proposed positioning method in this work achieves a positioning accuracy of 3–5 cm. Furthermore, the positioning accuracy was uniformly distributed in the time of one hour and unaffected by the initial position error.

**Figure 10:**
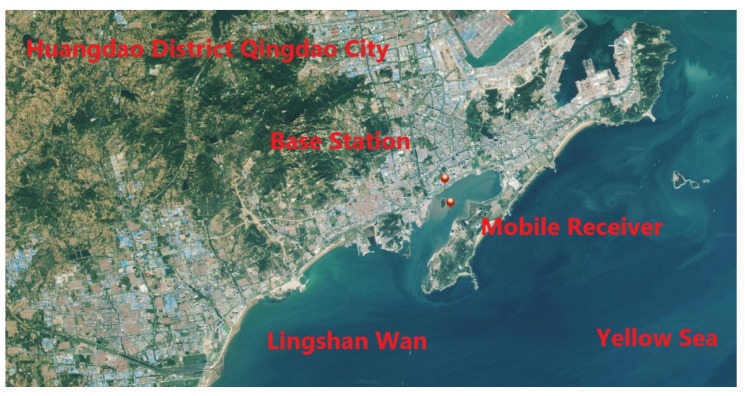
Positions of base station and mobile receiver for dynamic test carried on 23 September 2019 at 03:30–04:30 (GPS time).

**Figure 11:**
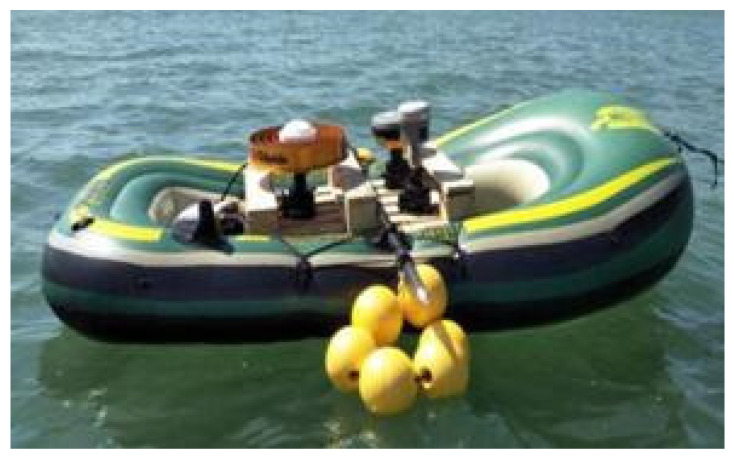
Dynamic test carried out by using Trimble R10 receiver on an inflatable boat.

**Figure 12:**
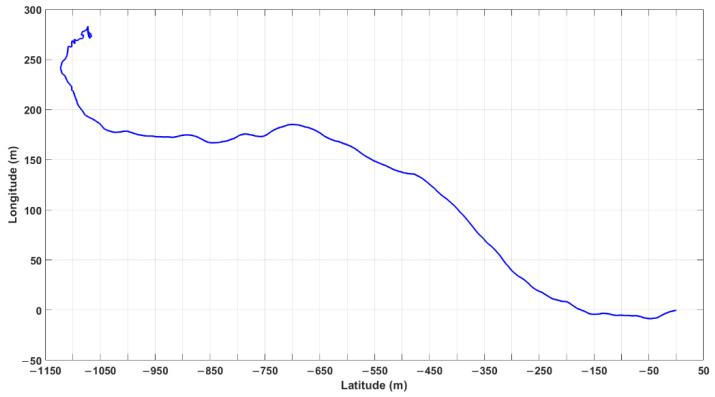
Trajectories of the mobile receiver, mounted on an inflatable boat and able to receive differential correction signals from the base station placed in Qingdao, Shandong province on 23 September 2019, at 03:30–04:30 (GPS time).

In order to confirm the positioning accuracy of the proposed positioning method, a data set collected in Qingdao, Shandong province, during a dynamic test carried out by means of a satellite receiver on an inflatable boat over one hour on 23 September 2019 was processed. A base station was placed near the mobile receiver, so that the RTK position data obtained by using GPS/Galileo/BeiDou satellites were used as reference ones. The positions of the base station and mobile receiver are shown in Figure 10. A Trimble R10 receiver was used as mobile receiver (Figure 11). Figure 12 indicates the trajectory of the mobile receiver, comprising RTK position data. The maximum distance between base station and mobile receiver is about 1300 m.

**Figure 13:**
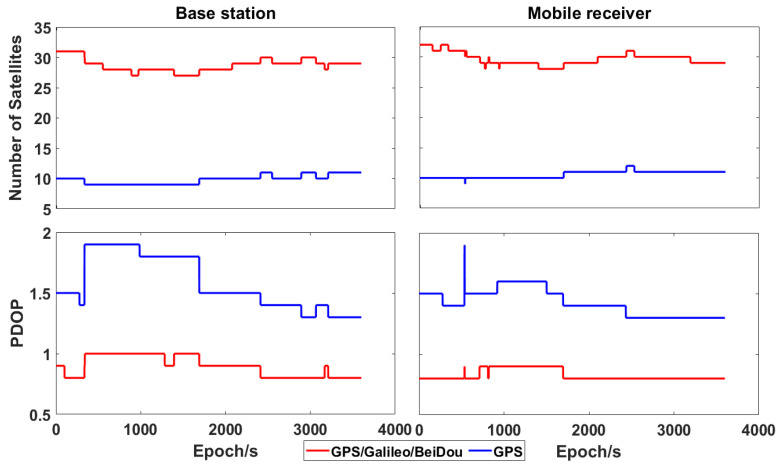
Number of visible satellites and PDOP related to base station and mobile receiver.

The number of visible satellites and PDOP related to both base station and mobile receiver are shown in Figure 13, which is similar to the results of static test: the combination of GPS, Galileo and BeiDou significantly increases the redundancy and improves the geometrical distribution of satellites (more uniform).

**Figure 14:**
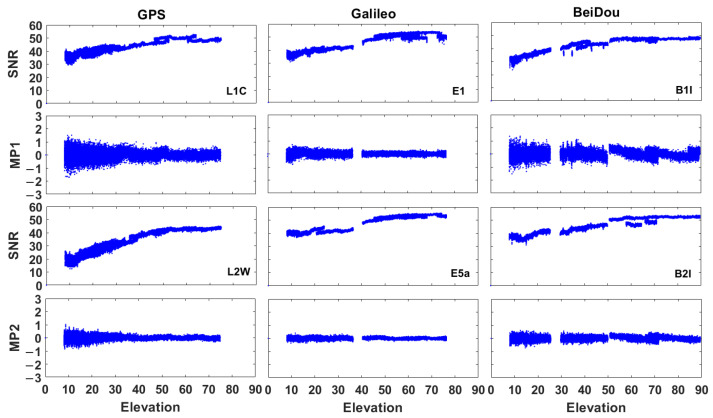
SNR (expressed as dB-Hz) and pseudorange multipath (MP1 and MP2, expressed as metres, the elevation was expressed in degrees) at mobile receiver by using GPS, Galileo and BeiDou satellites.

The SNR and pseudorange multipath (MP1/MP2) at mobile receiver are shown in Figure 14. The severe interferences were observed both for SNR measurements and MP1/MP2 by using GPS, Galileo and BeiDou satellites. These might result from sea surface reflection.

**Figure 15:**
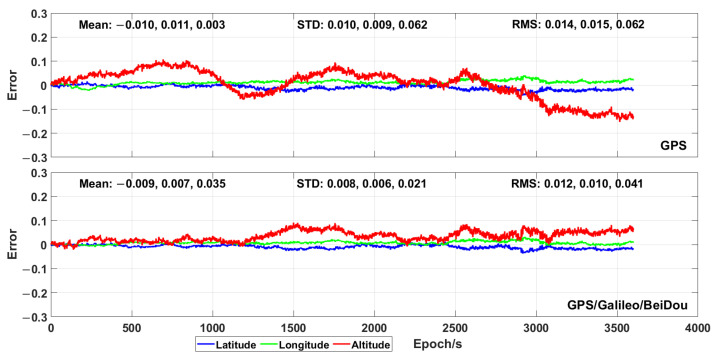
Positioning error obtained by means of the proposed time-relative positioning method during the dynamic test.

The results of the proposed positioning algorithm using only GPS and GPS/Galileo/BeiDou, respectively, are shown in Figure 15. By using only the GPS measurements, a positioning error (expressed as RMS) of 0.014, 0.015 and 0.062 m was achieved for longitude, latitude and altitude, respectively, while the maximum positioning errors were 0.044, 0.041 and 0.153 m, respectively. The positioning accuracy was also highly improved by the combination of GPS, Galileo and BeiDou. The positioning error, expressed as RMS, in one hour, was 0.012, 0.010 and 0.041 m for longitude, latitude and altitude, respectively. Meanwhile, the maximum positioning error and the standard deviation were much lower than those obtained by using only GPS for the three geodetic coordinates. The mean positioning error obtained by using multi-GNSS was also lower than that recorded by means of only GPS for the longitude and the latitude. Yet, a positioning error for the altitude, caused by unknown factors, resulted in a much higher mean value.

**Figure 16:**
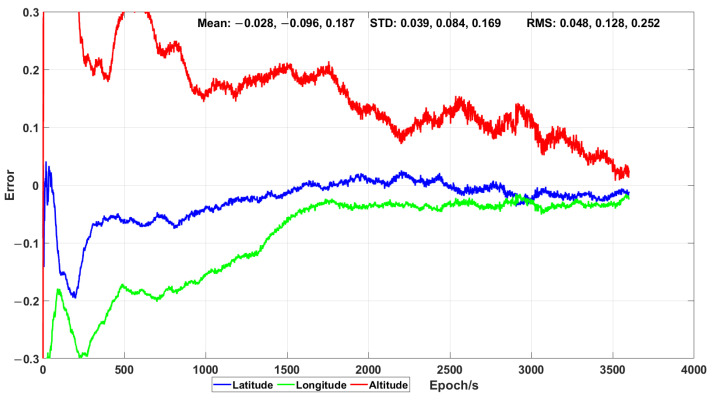
Positioning error (expressed in metres) obtained by means of PPP during the dynamic test.

The results of the traditional PPP technique using the CNT products and the combination of GPS, Galileo and BeiDou are shown in Figure 16. As expected, the initialisation time was also needed and the positioning accuracy (expressed as mean, standard deviation and RMS) was worse than that obtained by using the algorithm proposed in this work.

## 5. Conclusions

The real-time high accuracy position data are usually provided by RTK/NRTK or PPP/PPP-RTK techniques, based on GNSS. Yet, both RTK/NRTK and PPP/PPP-RTK have their disadvantages. In fact, The RTK/NRTK is very complex to use; besides the need for a reference station, the differential correction signals should be transmitted from the base station to the mobile receiver through two radio modems (i.e., a UHF transmitter, connected to the base station, and a UHF receiver, connected to the mobile receiver). Though the user position can be also determined by means of a single receiver by using the PPP or PPP-RTK technique, long initialisation time is needed for PPP. The above radio modems are needed also for the PPP-RTK technique.

The time-relative positioning based on TDCP overcomes the above issues and simplifies the ambiguities processing issue. Yet, it is difficult to achieve high positioning accuracy by using the traditional time-relative positioning for a long period. Furthermore, the positioning accuracy is significantly affected by the initial position error.

In order to overcome these issues, an improved time-relative positioning algorithm is proposed in this paper. The described algorithm uses the PPP model to estimate the parameters of current epoch, including position vector and ambiguities based on IF measurements. Then the estimated float IF ambiguities are used as constraints to estimate the position vector of the base epoch. Thus, the relative position between the base and the current epochs can be obtained by differencing the position vector between the base and the current epochs. As there is no approximation, differently from the traditional time-relative positioning method, the positioning accuracy is not affected by the initial position error. Meanwhile, the RTS products are used to constrain the satellite orbits and clocks, so that a high positioning accuracy is expected to be achieved. As described in previous paragraph, we considered the GNSS measurements to be free from cycle slips. In this work, both the Melbourne–Wübbena and geometry-free combination method and a robust Kalman filter were used to overcome this issue.

The numerical results obtained during static and dynamic tests show that the proposed time-relative positioning method can achieve centimetre-level horizontal and vertical positioning accuracy by using both only GPS and GPS/Galileo/BeiDou in the time of one hour. As expected, the positioning accuracy is significantly improved by the combination of GPS, Galileo and BeiDou, so that the horizontal and vertical accuracy, expressed as RMS is about 1–3 cm and 2–5 cm, respectively. The simulation results also confirm that the positioning accuracy of the proposed method is not affected by the initial position error. Due to the outstanding performance, the positioning method proposed in this paper can be used for long-period precise positioning by means of a single dual-frequency receiver. Furthermore, this method can be used in order to improve the positioning accuracy of the PPP technique, above all during the re-initialisation time.

## Figures and Tables

**Table 1:** Statistical results (expressed in metres) obtained by using only GPS.

		Mean			STD			RMS	
Time (h)	Latitude	Longitude	Altitude	Latitude	Longitude	Altitude	Latitude	Longitude	Altitude
0	0.006	0.026	−0.056	0.005	0.015	0.039	0.008	0.029	0.069
1	0.026	0.019	0.026	0.018	0.016	0.062	0.031	0.025	0.067
2	0.037	−0.021	0.009	0.018	0.020	0.046	0.041	0.029	0.047
3	−0.012	−0.001	−0.037	0.011	0.020	0.028	0.016	0.020	0.046
4	−0.002	−0.017	−0.013	0.023	0.017	0.048	0.023	0.024	0.050
5	0.036	−0.002	−0.005	0.017	0.015	0.044	0.039	0.015	0.044
6	−0.069	0.001	0.011	0.031	0.012	0.036	0.076	0.012	0.038
7	0.016	0.006	0.017	0.014	0.023	0.063	0.021	0.024	0.065
8	−0.006	−0.015	−0.053	0.010	0.023	0.046	0.012	0.027	0.070
9	−0.014	0.000	−0.008	0.012	0.007	0.030	0.018	0.007	0.031
10	0.011	−0.014	−0.010	0.013	0.014	0.020	0.017	0.020	0.022
11	−0.007	0.000	−0.010	0.005	0.008	0.047	0.009	0.008	0.048
12	−0.010	0.006	−0.016	0.010	0.007	0.023	0.014	0.009	0.028
13	−0.018	0.016	0.023	0.023	0.017	0.042	0.029	0.023	0.048
14	0.007	0.000	−0.054	0.010	0.021	0.059	0.012	0.021	0.080
15	0.010	0.009	0.018	0.024	0.011	0.080	0.026	0.014	0.082
16	−0.023	0.013	−0.083	0.020	0.015	0.067	0.030	0.020	0.107
17	0.004	0.021	−0.003	0.011	0.025	0.051	0.012	0.033	0.051
18	−0.037	0.003	−0.024	0.028	0.016	0.076	0.046	0.016	0.080
19	−0.024	−0.032	0.027	0.016	0.024	0.036	0.029	0.040	0.045
20	0.076	0.009	0.086	0.030	0.025	0.080	0.081	0.027	0.118
21	0.004	−0.002	0.006	0.004	0.011	0.035	0.006	0.011	0.036
22	0.026	0.017	−0.026	0.029	0.023	0.028	0.040	0.029	0.039
23	−0.016	0.003	−0.020	0.011	0.012	0.025	0.019	0.012	0.032

**Table 2:** Statistical results (expressed in metres) obtained by using GPS/Galileo/BeiDou.

		Mean			STD			RMS	
Time (h)	Latitude	Longitude	Altitude	Latitude	Longitude	Altitude	Latitude	Longitude	Altitude
0	0.010	−0.004	−0.048	0.005	0.009	0.021	0.011	0.010	0.052
1	0.007	−0.013	0.003	0.006	0.011	0.012	0.009	0.017	0.013
2	−0.008	0.030	0.027	0.007	0.010	0.024	0.011	0.031	0.036
3	−0.037	0.000	−0.006	0.020	0.017	0.025	0.042	0.017	0.026
4	0.013	−0.007	−0.004	0.025	0.008	0.030	0.028	0.011	0.030
5	0.029	0.015	−0.004	0.016	0.008	0.018	0.033	0.017	0.019
6	−0.023	0.004	−0.001	0.010	0.008	0.024	0.025	0.009	0.024
7	0.021	0.007	0.014	0.010	0.012	0.019	0.023	0.014	0.024
8	0.005	0.016	−0.015	0.008	0.008	0.019	0.010	0.018	0.024
9	−0.012	−0.001	−0.007	0.010	0.010	0.014	0.016	0.010	0.016
10	0.004	−0.027	0.003	0.011	0.016	0.016	0.012	0.032	0.017
11	−0.026	−0.015	0.002	0.012	0.010	0.018	0.029	0.018	0.018
12	−0.006	−0.009	−0.015	0.005	0.011	0.022	0.008	0.014	0.027
13	−0.016	0.024	−0.035	0.015	0.012	0.027	0.022	0.027	0.044
14	0.019	0.006	−0.015	0.010	0.009	0.018	0.022	0.011	0.024
15	0.002	0.006	0.035	0.010	0.006	0.024	0.010	0.009	0.042
16	−0.015	0.002	−0.034	0.014	0.006	0.022	0.021	0.006	0.040
17	−0.005	−0.004	−0.021	0.010	0.009	0.030	0.012	0.010	0.037
18	−0.031	0.021	−0.034	0.024	0.017	0.016	0.040	0.027	0.038
19	0.005	−0.014	0.005	0.010	0.011	0.029	0.011	0.017	0.029
20	0.058	−0.042	−0.016	0.018	0.015	0.047	0.061	0.045	0.050
21	0.008	0.002	−0.013	0.004	0.005	0.010	0.009	0.005	0.016
22	0.000	−0.003	−0.014	0.005	0.007	0.010	0.005	0.008	0.017
23	−0.006	0.006	0.012	0.005	0.005	0.027	0.008	0.008	0.029

## Data Availability

All data included in this study are available upon request by contact with the corresponding author.

## References

[1] Teunissen P.J.G., Khodabandeh A. (2015). Review and principles of PPP-RTK methods. J. Geod..

[2] Håkansson M., Jensen A.B., Horemuz M., Hedling G. (2017). Review of code and phase biases in multi-GNSS positioning. GPS Solut..

[3] Zumberge J.F., Heflin M.B., Jefferson D.C., Watkins M.M., Webb F.H. (1997). Precise point positioning for the efficient and robust analysis of GPS data from large networks. J. Geophys. Res. Solid Earth.

[4] Brack A., Männel B., Schuh H. (2020). GLONASS FDMA data for RTK positioning: A five-system analysis. GPS Solut..

[5] Li J., Yang Y., He H., Guo H. (2020). Benefits of BDS-3 B1C/B1I/B2a triple-frequency signals on precise positioning and ambiguity resolution. GPS Solut..

[6] Psychas D., Verhagen S. (2020). Real-time PPP-RTK performance analysis using ionospheric corrections from multi-scale network configurations. Sensors.

[7] Teunissen P., Odijk D., Zhang B. (2010). PPP-RTK: Results of CORS network-based PPP with integer ambiguity resolution. J. Aeronaut. Astronaut. Aviat. Ser. A.

[8] Dai L., Eslinger D., Sharpe T. Innovative algorithms to improve long range RTK reliability and availability. Proceedings of the ION NTM.

[9] Pan L., Gao X., Hu J., Ma F., Zhang Z., Wu W. (2020). Performance assessment of real-time multi-GNSS integrated PPP with uncombined and ionospheric-free combined observables. Advances in Space Research.

[10] Odijk D., Khodabandeh A., Nadarajah N., Choudhury M., Zhang B., Li W., Teunissen P.J. (2016). PPP-RTK by means of S-system theory: Australian network and user demonstration. J. Spat. Sci..

[11] Li Z., Chen W., Ruan R., Liu X. (2020). Evaluation of PPP-RTK based on BDS-3/BDS-2/GPS observations: A case study in Europe. GPS Solut..

[12] Zhao Y. (2017). Applying time-differenced carrier phase in nondifferential GPS/IMU tightly coupled navigation systems to improve the positioning performance. IEEE Trans. Veh. Technol..

[13] Michaud S., Santerre R. (2001). Time-relative positioning with a single civil GPS receiver. GPS Solut..

[14] Balard N., Santerre R., Cocard M., Bourgon S. (2006). Single GPS receiver time-relative positioning with loop misclosure corrections. GPS Solut..

[15] Ulmer K., Hwang P., Disselkoen B., Wagner M. Accurate azimuth from a single PLGR + GLS DoD GPS receiver using time relative positioning. Proceedings of the 8th International Technical Meeting of the Satellite Division of The Institute of Navigation (ION GPS 1995).

[16] Traugott J. (2010). Precise Flight Trajectory Reconstruction Based on Time-Differential GNSS Carrier Phase Processing. Ph.D. Thesis.

[17] Liu Z., Ji S., Chen W., Ding X. (2013). New fast precise kinematic surveying method using a single dual-frequency GPS receiver. J. Surv. Eng..

[18] Ji S., Sun Z., Weng D., Chen W., Wang Z., He K. (2019). High-precision Ocean navigation with single set of BeiDou short-message device. J. Geod..

[19] Yu W., Ding X., Chen W., Dai W., Yi Z., Zhang B. (2019). Precise point positioning with mixed use of time-differenced and undifferenced carrier phase from multiple GNSS. J. Geod..

[20] Wendel J., Meister O., Monikes R., Trommer G.F. Time-differenced carrier phase measurements for tightly coupled GPS/INS integration. Proceedings of the IEEE/ION PLANS.

[21] Soon B.K., Scheding S., Lee H.K., Lee H.K., Durrant-Whyte H. (2008). An approach to aid INS using time-differenced GPS carrier phase (TDCP) measurements. GPS Solut..

[22] Suzuki T. (2020). Time-relative RTK-GNSS: GNSS loop closure in pose graph optimization. IEEE Robot. Autom. Lett..

[23] Sun R., Cheng Q., Junhui W. (2020). Precise vehicle dynamic heading and pitch angle estimation using time-differenced measurements from a single GNSS antenna. GPS Solut..

[24] Colosimo G., Crespi M., Mazzoni A. (2011). Real-time GPS seismology with a stand-alone receiver: A preliminary feasibility demonstration. J. Geophys. Res. Solid Earth.

[25] Li X., Ge M., Guo B., Wickert J., Schuh H. (2013). Temporal point positioning approach for real-time GNSS seismology using a single receiver. Geophys. Res. Lett..

[26] Han S., Rizos C. (1996). Centimeter GPS kinematic or rapid static surveys without ambiguity resolution. Surv. Land Inf. Syst..

[27] Olynik M., Petovello M.G., Cannon M.E., Lachapelle G. Temporal variability of GPS error sources and their effect on relative positioning accuracy. Proceedings of the Institute of Navigation National Technical Meeting.

[28] Hadas T., Bosy J. (2015). IGS RTS precise orbits and clocks verification and quality degradation over time. GPS Solut..

[29] Abdelazeem M., Çelik R.N., El-Rabbany A. (2016). An enhanced real-time regional ionospheric model using IGS real-time service (IGS-RTS) products. J. Navig..

[30] Elsobeiey M., Al-Harbi S. (2016). Performance of real-time precise point positioning using IGS real-time service. GPS Solut..

[31] Lu Y., Wang Z., Ji S., Chen W. (2020). Assessing the positioning performance under the effects of strong ionospheric anomalies with multi-GNSS in Hong Kong. Radio Sci..

[32] Tregoning P., Herring T. (2006). Impact of a priori zenith hydrostatic delay errors on GPS estimates of station heights and zenith total delays. Geophys. Res. Lett..

[33] Saastamoinen J. (1972). Contributions to the theory of atmospheric refraction. Bull. Géodésique (1946–1975).

[34] Li X., Zhang X., Ren X., Fritsche M., Wickert J., Schuh H. (2015). Precise positioning with current multi-constellation global navigation satellite systems: GPS, GLONASS, Galileo and BeiDou. Sci. Rep..

[35] Hadas T., Teferle F.N., Kazmierski K., Hordyniec P., Bosy J. (2016). Optimum stochastic modeling for GNSS tropospheric delay estimation in real-time. GPS Solut..

[36] Shi J., Gao Y. (2014). A comparison of three PPP integer ambiguity resolution methods. GPS Solut..

[37] Shi J., Yuan X., Cai Y., Wang G. (2017). GPS real-time precise point positioning for aerial triangulation. GPS Solut..

[38] Liu Z. (2011). A new automated cycle slip detection and repair method for a single dual-frequency GPS receiver. J. Geod..

[39] Deo M., El-Mowafy A., Professor A. Cycle slip and clock jump repair with multi-frequency multi-constellation GNSS data for precise point positioning. Proceedings of the IGNSS Symposium.

[40] Zhang H., Ji S., Wang Z., Chen W. (2018). Detailed assessment of GNSS observation noise based using zero baseline data. Adv. Space Res..

[41] Zhou F., Dong D., Li W., Jiang X., Wickert J., Schuh H. (2018). GAMP: An open-source software of multi-GNSS precise point positioning using undifferenced and uncombined observations. GPS Solut..

